# Symptomatic stratification based on morphological features of carotid web (SCORE-WEB)

**DOI:** 10.3389/fneur.2026.1821579

**Published:** 2026-05-08

**Authors:** Sávio Batista, Mateus Damiani Monteiro, Fadi Nahab, Pedro Nascimento Martins, Jaydevsinh N. Dolia, Theja Yelam, Michael Frankel, John Oshinski, Ghada A. Mohamed, Jason Allen, Diogo C. Haussen

**Affiliations:** 1 School of Medicine, Emory University, Atlanta, GA, United States; 2 Department of Neurology, Grady Memorial Hospital, Atlanta, GA, United States; 3 Department of Neurology, Emory University Hospital, Atlanta, GA, United States

**Keywords:** carotid web (CaW), fibromuscular dysplasia (FMD), morphology, score, stroke

## Abstract

**Introduction:**

Carotid web (CaW) is an underrecognized variant of fibromuscular disease increasingly implicated in cryptogenic ischemic stroke. Several morphologic features, including lesion length, proximal angulation, and pocket size, have been associated with symptomatic presentation. However, no pragmatic prediction score has been validated to stratify asymptomatic patients.

**Methods:**

We performed a cross-sectional analysis of a prospectively maintained database of CaW cases collected between 2014 and July 2025 at two comprehensive stroke centers. Lesions were classified as symptomatic if associated with stroke or TIA involving the lesion vascular territory, and as asymptomatic if incidentally detected. Morphologic parameters extracted from computed tomography angiography (CTA) included pre-web angle, pocket area, lesion length, carotid bulb and distal cervical internal carotid caliber. A multivariable logistic regression model was developed, and a simplified score (SCORE-WEB) was derived based on cutoffs across these variables. Discriminative performance was assessed through ROC curves.

**Results:**

A total of 126 CaW were analyzed, including 80 symptomatic and 46 asymptomatic cases (30 incidental and 16 contralateral to a symptomatic CaW). Symptomatic lesions had more acute pre-web angles, larger pocket areas, and greater distal cervical internal carotid and bulb calibers compared with asymptomatic lesions, while substantial overlap in web length was observed. The multivariable logistic regression model demonstrated good discriminative performance for symptomatic presentation (AUC 0.803). The simplified SCORE-WEB (ranging from 0–11) achieved an AUC of 0.824. The Youden Index (J = 0.51) identified an optimal cutoff of ≥7 points, yielding a sensitivity of 0.54, specificity 0.97, positive predictive value 97.4%, and negative predictive value 44.4%.

**Conclusion:**

The constellation of distinct morphological features appear to collectively and significantly determine the symptomatic status of CaW. The simplified SCORE-WEB, derived from five anatomical parameters, showed good discrimination and potential practical applicability. These findings warrant validation in larger and prospective datasets. The role of clinical variables in enhancing predictive performance merits further investigation.

## Introduction

Carotid web (CaW) is an underrecognized variant of fibromuscular disease increasingly implicated in cryptogenic ischemic stroke (1). Although its association with thromboembolism has strengthened in recent years, CaW are sometimes identified incidentally (2). The likelihood of asymptomatic CaW progressing to a stroke are not well understood but the overall risks appear to be relatively low (3).

Several morphologic characteristics of CaW have been linked to symptomatic status, including increased lesion length, greater web volume, and more acute proximal angulation (4–8). These factors may enhance flow hemodynamic disturbances, increasing the risk of thrombogenesis and embolism. Computational fluid dynamics and 4-D Flow MRI studies have corroborated these conceptual insights (4, 5), demonstrating that CaW induces large areas of flow recirculation, low wall shear stress and high oscillatory shear index, parameters that themselves correlate with proximal angulation and the extent of endoluminal protrusion (4–8).

This study aimed to develop a pragmatic prediction stratification framework through the evaluation of a scoring system — SCORE-WEB — that incorporates key anatomical parameters from computed tomography angiography (CTA). The objective was to determine whether this approach could effectively discriminate between symptomatic and asymptomatic lesions. This predictive capability could provide a foundation for approaches aimed at identifying carotid webs with higher probability of symptomatic progression and ultimately inform clinical decision-making.

## Methods

This was a cross-sectional retrospective analysis derived from a prospectively maintained institutional database of patients diagnosed with CaW spanning July 2014 to July 2025 at two comprehensive stroke centers. Patients with symptomatic or asymptomatic CaW diagnosed via CTA imaging during evaluation for stroke, other neurological symptoms or prospectively found incidentally during clinical practice were included in the database (symptomatics since 2014 and asymptomatics 2016). Follow-up information was prospectively collected or obtained through institutional medical record review when available. The study was approved by the Emory University Institutional Review Board (IRB #00091421) and followed the STROBE (Strengthening the Reporting of Observational Studies in Epidemiology) guidelines. The requirement for written informed consent was waived due to the retrospective nature of the study. The research was conducted in accordance with the Declaration of Helsinki.

### CaW identification and classification

High-resolution CTA with thin-slice reconstructions (section interval ≤0.625 mm) was used to identify CaW, which were independently confirmed by two board-certified readers (the panel included a neuroradiologist and two neurointerventionists). CTA examinations (120 kVp) were acquired using multi-detector CT systems. At the first center, imaging was performed on GE Discovery 705HD or GE Revolution HD scanners (GE Healthcare, Waukesha, WI); at the second center, acquisitions were obtained using GE Revolution HD, GE Discovery CT750 HD, or Siemens SOMATOM Definition Edge (Siemens Healthineers, Washington, DC) scanners all standardized across the system. CaW were classified as symptomatic in patients with stroke or transient ischemic attack (TIA) referrable to the lesion vascular territory. In symptomatic cases, the presence of competing stroke mechanisms was documented and encompassed definitive/probable/possible alternative causes. Asymptomatic CaW encompasses incidental findings in patients without a stroke within its correspondent vascular territory and asymptomatic lesions contralateral to the symptomatic CaW. Given the potential biological differences between these subgroups, analyses were performed with a primary focus on incidental asymptomatic lesions for model development, while all asymptomatic lesions were included in sensitivity analyses. Additional information regarding the database has been previously reported (6). Image analysis was performed using Horos software (Horos Project, Geneva, Switzerland).

### Selection of morphological variables

Candidate morphologic variables were measured via CTA analysis and compared between the symptomatic and asymptomatic cohorts. Anatomical parameters were selected for model development based on prior evidence of their association with symptomatic presentation: lesion length has been shown to be associated with symptomatic CaW (7–10), as well as the pre-web angle and web pocket area (7–9, 11). The ratio of web length/carotid bulb (“web-to-bulb” ratio) diameter and the degree of stenosis (by NASCET criteria) have also been postulated to increase stroke risk (9, 12, 13). Considering that both NASCET and web-to-bulb ratio are determined by length, bulb size, and distal caliber, we opted to analyze the fundamental dimensions (carotid bulb diameter and mid-distal cervical internal carotid diameter, in addition to length, angle, pocket and area) rather than derivative indices. Other anatomical variables with significant differences within the present cohort (*p* < 0.05) were also included.

The methodology for the measurement of these variables has been previously published (14); in short, the analysis was based on the identification of the CaW ridge orientation and a projection exactly perpendicular to the web ridge using multiplanar reformat views was selected (Supplementary Figure 1A). Maximum-intensity projections (MIP) of 5 mm thickness were used for all measurements (Supplementary Figures 1B,C); the thickness could be adjusted for optimization of the lesion visualization if necessary. The length of the CaW was defined as the distance from the midpoint of the web base to its apex. After defining the longitudinal axis, the pocket area (corresponded to the region between the rostral surface of the web to a line drawn at 90° off the carotid axis) and the pre-web angle (angle formed between the line tangent to the caudal CaW surface and the longitudinal axis) were calculated. The bulb diameter represented the luminal diameter at the carotid bulb immediately distal to the web. The distal caliber was defined as the luminal diameter of the cervical internal carotid artery where walls are parallel. CTA image post-processing and quantitative measurements were centralized and conducted using uniform methodology.

### SCORE-WEB design

We derived a simplified morphological prediction score (SCORE-WEB) based on the five selected anatomical parameters. Each variable was discretized into one to five clinically interpretable strata. Cutoffs were derived by examining the distribution of symptomatic cases across finely spaced intervals (length: 0.1-mm steps; area: 0.5-mm^2^ steps; angle: 1° steps). Thresholds and points were defined at points where the symptomatic rate changed meaningfully, then consolidated and rounded to clinically practical values. Adjacent intervals were merged as needed to ensure a monotonic trend (increasing or decreasing) across categories, facilitating consistent interpretation and clinical applicability. The resulting SCORE-WEB ranges from 0 to 11 points, with higher scores reflecting greater symptomatic presentation.

### Bae et al. scoring system

Bae et al. proposed a morphological score to predict the likelihood of symptomatic presentation in CaW cases (13). The score incorporated two parameters: stenosis degree (NASCET method) and pre-web angle. Each 10% increment in stenosis (20–80%) and smaller angles (10°–70°) were assigned progressively higher points, summed to a total score. A cutoff of ≥8 points was suggested to identify symptomatic CaW. We applied this scoring system to our cohort for external validation.

### Statistical analysis

The group with incidental asymptomatic CaW was selected for model development. Group comparisons between symptomatic and asymptomatic patients were performed using the Mann–Whitney U test for continuous variables and either the chi-square or Fisher’s exact test for categorical variables, as appropriate. To assess measurement reliability, two independent raters (SRB and DCH) performed morphological measurements blinded to the clinical information in randomly selected subset of 20 consecutive patients with unilateral CaW, followed by single rater reads. Interobserver agreement was quantified using the intraclass correlation coefficient (ICC), calculated using a two-way random-effects model for absolute agreement. Discriminative performance of both the multivariable logistic model and the simplified score was evaluated using the area under curve (AUC) of the receiver operating characteristic (ROC). As an exploratory analysis, clinical variables including age and a composite comorbidity burden (dyslipidemia, smoking, atrial fibrillation, hypertension, and diabetes mellitus) were added to the model to assess whether they improved performance. Predicted probabilities were generated by mapping the total score points using logistic regression. Calibration of the score was evaluated by comparing predicted probabilities with observed event rates across score values and graphically assessed using calibration plots. To determine the optimal discriminative threshold, a Youden Index (J = sensitivity + specificity − 1) was calculated for each possible cutoff value. Internal validation was performed using bootstrap resampling to estimate optimism-corrected performance metrics. To assess the robustness of the SCORE-WEB in a more etiologically homogeneous population, a sensitivity analysis was performed after excluding patients with competing stroke etiologies. All statistical analyses were performed in R software (version 4.2.2), and a *p*-value < 0.05 was considered statistically significant.

## Results

A total of 126 CaW were included, comprising 80 symptomatic and 46 asymptomatic lesions (contralateral to symptomatic or incidental). The comparison of symptomatic with all asymptomatic CaW lesions is shown on Table 1.

**Table 1:** Clinical and morphological characteristics of both symptomatic and asymptomatic carotid web (CaW) cohorts.

Variable	Symptomatic (*n* = 80)	Asymptomatic unilateral (*n* = 30)	*p* vs. symp (unilateral)	Asymptomatic all (*n* = 46)	*p* vs. symp (all)
Clinical characteristics					
Age ± SD	50.4 ± 11.0	51.1 ± 16.7	0.892	–	–
Female (%)	53 (66.3%)	15 (50.0%)	0.337	–	–
Race (%)					
White	7/75 (9.3%)	1/26 (3.8%)	0.443	–	–
Black or African American	67/75 (89.3%)	19/26 (73.1%)	0.036	–	–
Asian	1/75 (1.3%)	0/26 (0.0%)	1.000	–	–
Other	0/75 (0.0%)	6/26 (23.1%)	0.0003	–	–
Comorbidities					
Hypertension	44 (55.0%)	15 (50.0%)	1.0	–	–
Diabetes mellitus	13 (16.3%)	6 (20.0%)	0.565	–	–
Dyslipidemia	14 (17.5%)	10 (33.3%)	0.060	–	–
Atrial fibrillation	2 (2.6%)	2 (6.7%)	0.59	–	–
Smoker	14 (17.5%)	7 (23.3%)	0.406	–	–
Morphological characteristics (median, IQR)					
Proximal web angle	33.3° (24.4–43.5)	48.7° (36.5–56.4)	**<0.01**	41.7° (34.3–63.0)	**0.022**
Area pocket	1.47mm^2^ (0.88–2.54)	0.94mm^2^ (0.00–1.53)	**0.024**	0.95mm^2^ (0.22–1.46)	**0.013**
Length	2.55 mm (2.15–3.43)	2.5 mm (1.97–2.87)	0.184	2.29 mm (1.80–2.87)	**0.034**
Bulb caliber	7.8 mm (7.30–8.30)	7.30 mm (6.30–8.00)	**0.05**	7.45 mm (6.53–8.17)	0.240
Distal caliber	4 mm (3.60–4.30)	3.65 mm (3.40–4.00)	**0.008**	3.85 mm (3.42–4.00)	**0.017**
Web-bulb ratio	4.20 (3.50–4.90)	3.60 (3.12–4.00)	**0.041**	3.80 (3.25–4.90)	0.398
Base	2.74 mm (2.04–3.97)	2.53 mm (2.10–3.35)	0.63	2.49 mm (2.02–3.35)	0.379
NASCET stenosis (median, IQR)	2% (0–17%)	0% (0–8%)	0.049	0% (0–17%)	0.608
NASCET categories					
0–29%	74 (92.5%)	29 (96.7%)	0.672	38 (82.6%)	0.138
30–70%	6 (7.5%)	1 (3.3%)	0.672	7 (15.2%)	0.223
>70%	0 (0.0%)	0 (0.0%)	1.0	1 (2.2%)	0.362

When restricting asymptomatic lesions to patients with incidental CaW (*n* = 30), the findings were consistent. Symptomatic cases showed a more acute pre-web angle (median 33.3°, IQR 24.4–43.5 vs. 48.7°, IQR 36.5–56.4), larger pocket area (median 1.47mm^2^, IQR 0.88–2.54 vs. 0.94 mm^2^, IQR 0.00–1.53), larger distal cervical internal carotid caliber (median 4 mm, IQR 3.60–4.30 vs. 3.65 mm, IQR 3.40–4.00), as well as bulb caliber (median 7.8 mm, IQR 7.30–8.30 vs. 7.30 mm, IQR 6.30–8.00). Significant overlap between web length was noticed between symptomatic (median 2.55 mm, IQR 2.15–3.43) and asymptomatic (2.5 mm, IQR 1.97–2.87) webs. Nine patients (11.2%) with symptomatic CaW had competing etiology (Supplementary Table 1). Other clinical and morphological characteristics are detailed in Table 1. The ICC demonstrated excellent agreement for the pre-web angle (ICC = 0.97) and pocket area (ICC = 0.98), with good agreement for web length (ICC = 0.76), carotid bulb caliber (ICC = 0.83), cervical internal carotid caliber (ICC = 0.71), perimeter (ICC = 0.81), distal-web angle (ICC = 0.75), and base (ICC = 0.71). The reasons for imaging in asymptomatic web patients are depicted on the Supplementary Table 2.

### Multivariable logistic model

**Figure 1:**
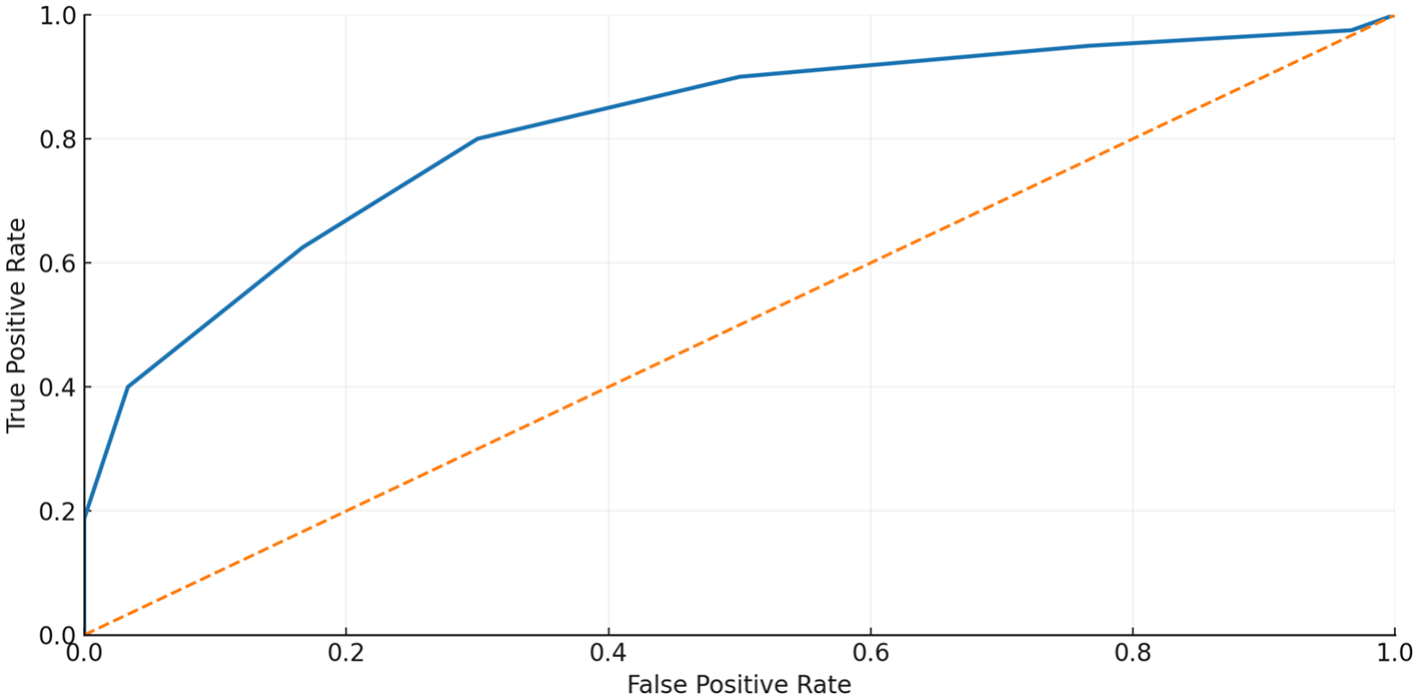
ROC analysis of the multivariable model with selected coefficients—pre-web angle, pocket area, lesion length, bulb caliber, and distal caliber.

The selected morphological variables were tested in the multivariable logistic model that analyzed symptomatic (*n* = 80) and unilateral incidental (*n* = 30) CaW, and the coefficients for predicting symptomatic presentation were: −0.041 per increment of 1° of the pre-web angle, +0.154 per 1mm^2^ of area pocket, +0.385 per 1 mm of CaW length, −0.147 per 1 mm of bulb, and +0.829 per 1 mm of the caliber distal. ROC curve analysis of these predicted probabilities demonstrated good discrimination between symptomatic and asymptomatic carotid webs, with an AUC of 0.803 (Figure 1). The addition of clinical variables, including age and a composite comorbidity burden (dyslipidemia, smoking, atrial fibrillation, hypertension, and diabetes mellitus), did not meaningfully improve model performance (AUC 0.810).

### SCORE-WEB

**Figure 2:**
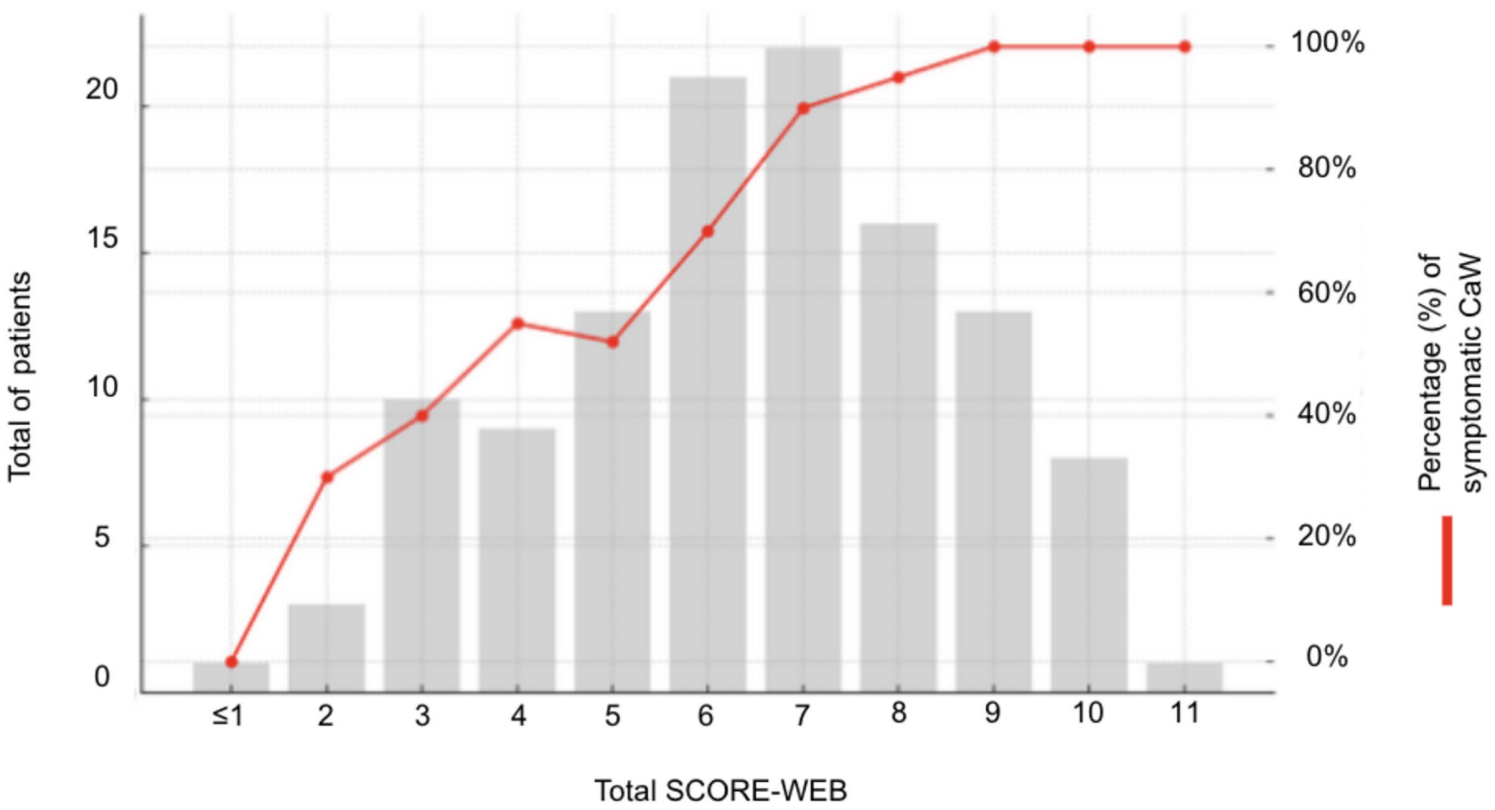
Distribution of SCORE-WEB values and corresponding frequency of symptomatic webs. The SCORE-WEB (simplified morphological prediction score) distribution is depicted in gray bars, while the observed rates of symptomatic presentation are demonstrated by the red line.

The design of the simplified SCORE-WEB, including cutoffs and assigned points, is presented in Table 2. When applied to our cohort of symptomatic and asymptomatic/incidental CaW, the SCORE-WEB demonstrated strong discriminative performance in differentiating the groups, with an AUC of 0.824 (Supplementary Figure 2). The distribution of patients across total score values, along with the observed proportion of symptomatic cases at each level, is shown in Figure 2. The proportion of symptomatic patients increased in a monotonic fashion across SCORE-WEB strata. The Youden Index (J = 0.51) was observed at a SCORE-WEB ≥7, which was therefore selected as the optimal cutoff. Based on this threshold, a SCORE-WEB ≥7 yielded a sensitivity of 0.54 and a specificity of 0.97 in this cohort. This threshold led to a positive predictive value (PPV) of 97.4% and a negative predictive value (NPV) of 44.4%, indicating strong rule-in performance for symptomatic CaW morphology. Conversely, rule-out thresholds for symptomatic CaW were as follows: ≥2 points, sensitivity 0.99 and specificity 0.10; and ≥1 point, sensitivity 1.00 and specificity 0.03.

**Table 2:** SCORE-WEB (simplified morphological prediction score).

Variable	Interval	Assigned point
Length (mm)	0–2	0
	>2–3	1
	>3	2
Pre-web angle (degree)	0–25	4
	>25–35	3
	>35–40	2
	>40–50	1
	> 50	0
Area pocket (mm^2^)	0–0.35	0
	>0.35–1	1
	>1	2
Bulb (mm)	0–7.5	1
	>7.5	0
Caliber distal (mm)	0–3.5	0
	>3.5–4	1
	> 4	2

Calibration analysis demonstrated good agreement between predicted probabilities derived from the SCORE-WEB and observed event rates across score values, as illustrated in the calibration plot (Supplementary Figure 2), with an overall Brier score of 0.15. Internal validation using bootstrap resampling (1,000 iterations) showed minimal optimism, with an optimism-corrected AUC of 0.806 and a Brier score of 0.16. The bootstrap-corrected calibration slope was 1.01, and the events-per-variable ratio was 16, supporting good calibration and limited evidence of overfitting. In a sensitivity analysis excluding patients with competing stroke etiologies, the SCORE-WEB demonstrated stable performance (AUC 0.793; Brier score 0.16). In contrast, when all asymptomatic lesions (including incidental and contralateral cases) were included, model discrimination decreased (AUC 0.697).

### Bae et al. score

When we applied the CaW score and cutoff proposed by Bae et al. to our cohort of symptomatic CaW, only 42.5% of patients had a score of ≥8. The distribution of the total of patients according to pre-web angle and stenosis rate can be seen in the Supplementary Figure 3.

## Discussion

Our findings suggest that symptomatic carotid webs are defined by a constellation of morphological features whose combined effect may influence clinical risk. A multivariable logistic regression model aggregating isolated phenotypical characteristics demonstrated strong discriminative performance. The simplified SCORE-WEB preserved accuracy while possibly offering better clinical applicability.

The identification of morphological features (and their interactions) that optimally discriminate between symptomatic and asymptomatic presentations may have important clinical implications. CaW lesion length reflects the longitudinal extent of the endoluminal fibrotic projection and was one of the first morphologic parameters linked to symptomatic presentation (10). The longer webs would generate enhanced flow disturbance, amplifying the zone of flow recirculation and ultimately thrombus formation (7–9, 11). Although prototypical shelf-like CaW lesions have similar pre and post angulation relative to the vessel axis (shelf-like phenotype), our experience is that morphology varies and therefore the overall correlation between the two angles may be attenuated. This may explain the observed stronger association between proximal angle with symptomatic status as compared to distal angle in our results. The web angle has been associated with stroke presentation (7–9, 11), higher turbulence intensity, as well as with larger areas of low wall shear stress distal to the web (predisposing to endothelial dysfunction) coupled with higher shear stress over the web proximal surface (leading to platelet activation) (13, 15), and the pre-web angle may have a significant role to these effects. The pocket area may function as the backward-facing step concept from fluid dynamics, where the addition of a step in a fluidic oscillator leads to a drop in pressure, flow separation and results in not only a primary but a secondary area of recirculation (16). Finally, the bulb caliber and the distal cervical internal carotid diameters may not individually have a significant impact, but are important for the model’s internal reference.

Our framework demonstrated good discrimination for symptomatic presentation, despite individual morphologic variables showing limited predictive value on their own. Single geometric features often share variance, and no individual parameter can fully inform the complexity of the flow environment. As with other vascular conditions such as cerebral aneurysms (where morphological features like size, morphology, daughter sac, location, and MRI wall enhancement, etc., influence rupture risk) (17) and arteriovenous malformations (in which associated aneurysms, deep or infratentorial location, exclusive deep venous drainage, single venous outflow, smaller nidus size, etc., increase hemorrhage risk) a more robust prediction assessment model in CaW may likewise require integrating multiple morphologic features (18, 19). Collectively, the five selected parameters may capture complementary aspects of CaW geometry. Their integration into SCORE-WEB provides a physiologically grounded and clinically practical framework that could serve as a foundation for further analyses.

We assessed the validity of the previously proposed CaW score by Bae et al. in the present cohort (13). The scoring system derived from computational fluid dynamics modeling and used both the degree of vessel stenosis generated by the CaW [NASCET criteria (13)] and the proximal lesion angle. Contrary to the original findings, which indicated that 97% of symptomatic cases presented scores ≥8, our analysis revealed that only 42.5% of our patients exhibited scores at or above this threshold. The original score assumed a simplified tubular bulb geometry and relied on published figures, meaning that patient selection was potentially biased towards more conspicuous webs and that measurements may not have been reliable due to the lack of standardization on image projections. While this approach demonstrates the impact of lesion length and angulation, it may not fully capture the natural carotid bulb/web anatomic variants. The NASCET-based stenosis grading may have limited applicability in the evaluation of CaW, as the residual lumen is generally not significantly narrowed relative to the distal internal carotid artery (10). This is reflected in the present symptomatic cohort, which demonstrated a median stenosis of 2% (IQR 0–17%), highlighting that carotid web-related stroke mechanisms may differ from atherosclerotic-derived events. The web-to-bulb ratio has been shown to be higher in patients with symptomatic presentation (9). This measurement has biological plausibility if the web is considered as a backward-facing step equivalent in terms of its impact on flow patterns at the level of the bulb (16). Indeed, our findings corroborated the web-to-bulb ratio as significantly different between symptomatic and incidental lesions. This suggests that the interplay between lesion length and bulb morphology may significantly influence the flow patterns, and justified the incorporation of the carotid bulb diameter (in addition to the web length) into the model.

Several limitations merit consideration. This was a double-center retrospective analysis, and external validation in larger, multicenter cohorts would be necessary to confirm generalizability. Although morphologic measurements were performed systematically, inter-rater variability and differences in imaging acquisition may affect reproducibility. Our analysis was restricted to anatomic features; integrating clinical variables or direct hemodynamic measures may further improve predictive accuracy. Demographic characteristics and comorbidities were not found to play a significant role in this cohort, but this may reflect selection bias, as patients with asymptomatic webs commonly had neurological symptoms and/or competing vascular risk factors that led to the indication of the vascular imaging study. Finally, although SCORE-WEB demonstrated good discriminative performance, its clinical role in guiding management decisions—particularly regarding antithrombotic therapy or revascularization—remains to be tested in prospective studies, especially considering the lack of systematic follow-up in asymptomatic patients. Given its relatively limited sensitivity, the score may function primarily as a rule-in rather than a screening tool; higher scores could theoretically support consideration of more intensive management, whereas lower scores should not be interpreted as low prediction. Until validated by further work, it should be applied cautiously and considered a research tool.

## Conclusion

The constellation of distinct morphological features appear to collectively and significantly determine the symptomatic status of CaW. The simplified SCORE-WEB, derived from five anatomical parameters, showed good discrimination and practical applicability. These findings warrant validation in larger and prospective datasets. The role of clinical variables in enhancing predictive performance merits further investigation.

## Data Availability

The raw data supporting the conclusions of this article will be made available by the authors, without undue reservation.

## References

[1] Liang S Qin P Xie L Niu S Luo J Chen F . The carotid web: current research status and imaging features. Front Neurosci. (2023) 17:1104212. doi: 10.3389/fnins.2023.1104212, 36860618 PMC9968728

[2] Ferreira F. M. Viana L. S. Batista S. Yelam T. Nogueira R. G. Al-Bayati A. R. . Incidental Carotid Webs in Trauma Patients, Clin Neurol Neurosurg. (2026) 263:109337. doi: 10.1016/j.clineuro.2026.109337 41637888

[3] Kim SJ Nogueira RG Haussen DC. Current understanding and gaps in research of carotid webs in ischemic strokes: a review. JAMA Neurol. (2019) 76:355–61. doi: 10.1001/jamaneurol.2018.3366, 30398546

[4] El Sayed R Lucas CJ Cebull HL Nahab FB Haussen DC Allen JW . Subjects with carotid webs demonstrate pro-thrombotic hemodynamics compared to subjects with carotid atherosclerosis. Sci Rep. (2024) 14:10092. doi: 10.1038/s41598-024-60666-7, 38698141 PMC11066020

[5] Ozaki D Endo T Suzuki H Sugiyama SI Endo K Itabashi R . Carotid web leads to new thrombus formation: computational fluid dynamic analysis coupled with histological evidence. Acta Neurochir. (2020) 162:2583–8. doi: 10.1007/s00701-020-04272-2, 32152755

[6] Landzberg D Nogueira RG Al-Bayati AR Kim SJ Bouslama M Pisani L . Baseline characteristics of patients with symptomatic carotid webs: a matched case control study. J Stroke Cerebrovasc Dis. (2021) 30:105823. doi: 10.1016/j.jstrokecerebrovasdis.2021.105823, 34034127

[7] Bala F Alhabli I Singh N Benali F Coutts S Goyal M . Relationship between carotid web morphology on CT angiography and stroke: a pooled multicenter analysis. Int J Stroke. (2024) 19:1046–52. doi: 10.1177/17474930241264141, 38877750

[8] Yirmibeş EÖB Şengeze N Gürel B. The frequency of carotid web in cryptogenic stroke and its association with stroke risk factors. J Stroke Cerebrovasc Dis. (2025) 34:108295. doi: 10.1016/j.jstrokecerebrovasdis.2025.108295, 40096924

[9] Tabibian BE Parr M Salehani A Mahavadi A Rahm S Kaur M . Morphological characteristics of symptomatic and asymptomatic carotid webs. J Neurosurg. (2022) 137:1727–32. doi: 10.3171/2022.2.JNS212310, 35426815

[10] Haussen DC Grossberg JA Bouslama M Pradilla G Belagaje S Bianchi N . Carotid web (intimal fibromuscular dysplasia) has high stroke recurrence risk and is amenable to stenting. Stroke. (2017) 48:3134–7. doi: 10.1161/STROKEAHA.117.019020, 29018133

[11] von Oiste GG Sangwon KL Chung C Narayan V Raz E Shapiro M . Use of carotid web angioarchitecture for stroke risk assessment. World Neurosurg. (2024) 182:e245–52. doi: 10.1016/j.wneu.2023.11.091, 38006939

[12] Bae T Ko JH Chung J. Turbulence intensity as an Indicator for ischemic stroke in the carotid web. World Neurosurg. (2021) 154:e443–57. doi: 10.1016/j.wneu.2021.07.049, 34325025

[13] Bartlett ES Walters TD Symons SP Fox AJ. Quantification of carotid stenosis on CT angiography. AJNR Am J Neuroradiol. (2006) 27:13–9. 16418349 PMC7976065

[14] Damiani Monteiro M Tarek MA Martins PN Allen JW Nogueira RG Landzberg D . Carotid web catheter angiography hemodynamic parameters. J. Neuroint. Surg. (2025) 17:843–7. doi: 10.1136/jnis-2024-021948, 39019504

[15] Kleeberg A Luft T Golkowski D Purrucker JC. Endothelial dysfunction in acute ischemic stroke: a review. J Neurol. (2025) 272:143. doi: 10.1007/s00415-025-12888-6, 39812851 PMC11735568

[16] Iskandar W Julian J Adhynugraha MI Hasim F Harinaldi H. Parametric study on the backward-facing step height in the mixing chamber of fluidic oscillator. J Appl Fluid Mech. (2025) 18:1205–16. doi: 10.47176/jafm.18.5.3100

[17] Ho AL Lin N Frerichs KU Du R. Intrinsic, transitional, and extrinsic morphological factors associated with rupture of intracranial aneurysms. Neurosurgery. (2015) 77:433–42. doi: 10.1227/NEU.0000000000000835, 26075307

[18] Koester SW Batista S Bertani R Yengo-Kahn A Roth S Chitale R . Angiographic factors leading to hemorrhage in AVMs: a systematic review and meta-analysis. Neurosurg Rev. (2023) 46:72. doi: 10.1007/s10143-023-01971-z, 36935466

[19] Kim H Nelson J McCulloch CE Hess C Hetts SW Flemming K . Risk of future hemorrhage from unruptured brain arteriovenous malformations: the multicenter arteriovenous malformation research study (MARS). JAMA Neurol. (2025) 82:e253581. doi: 10.1001/jamaneurol.2025.3581, 41051760 PMC12501859

