# Supplementary material for: Symptomatic stratification based on morphological features of carotid web (SCORE-WEB)
Source: Front Neurol. 2026 May 8;17:1821579. doi: 10.3389/fneur.2026.1821579 (PMC13193918; doi:10.3389/fneur.2026.1821579)
Supplement: Supplementary file 1 [file Data_Sheet_1.pdf]

Suppl Figure 1.: ROC analysis of the SCORE-WEB (simplified morphological risk score)

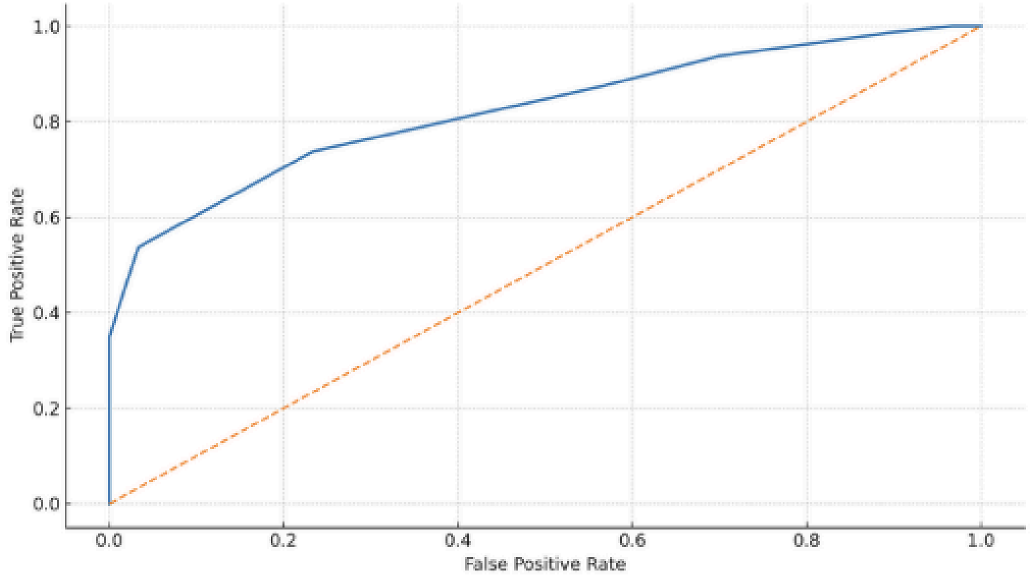

Suppl Figure 2.: Calibration Plot of the SCORE-WEB

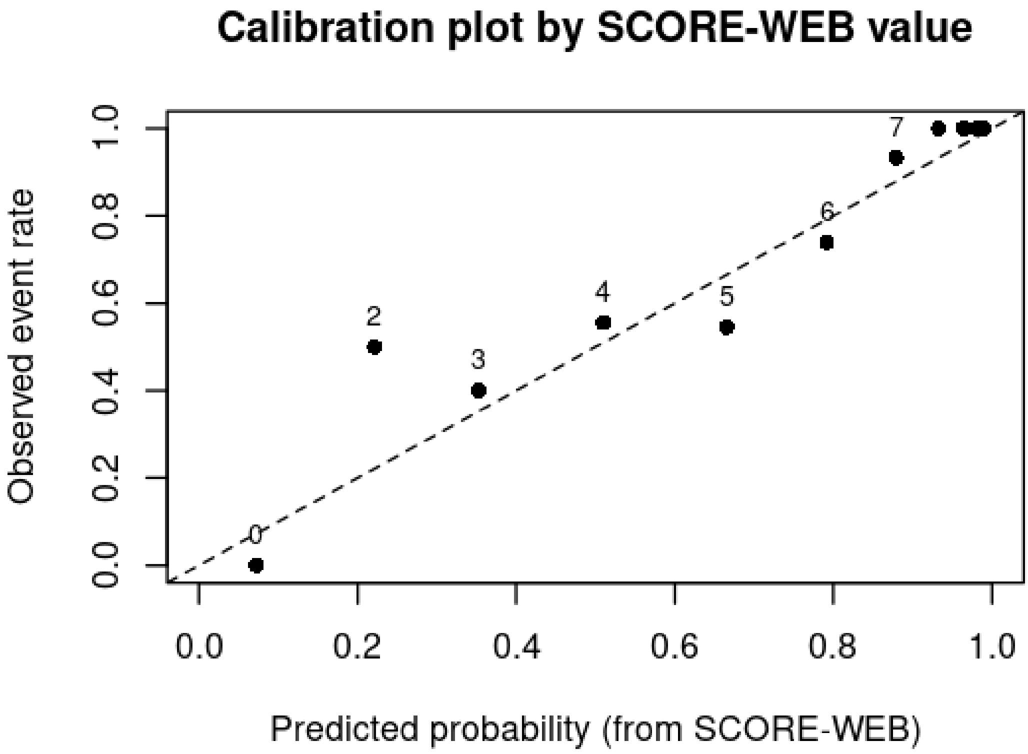

Suppl Figure 3.: Distribution of symptomatic carotid webs (CaW) from our cohort according to the score proposed by Bae et al., showing the number of patients allocated within each cell

	**Stenosis rate**	20%	30%	40%	50%	60%	70%	80%
**Angle**	**Score**	2	3	4	5	6	7	8
70°	2	4	0	0	0	0	0	0
60°	3	9	1	0	0	0	0	0
50°	4	12	4	0	0	0	0	0
40°	5	16	3	0	1	0	0	0
30°	6	11	2	1	1	0	1	0
20°	7	11	1	0	2	0	0	0
10°	8	0	0	0	0	0	0	0

Suppl Table 1.: Distribution of Competing Stroke Etiologies in Symptomatic Carotid Webs

**Competitive etiology**	
Cardioembolic - Atrial Fibrillation	2 (22.2%)
Cardioembolic - Other causes	5 (55.6%)
Hypercoagulable State	2 (22.2%)

Suppl Table 2.: Diagnosis/Indications for imaging in Asymptomatic Carotid Webs

**Indication of Vascular Imaging**	N=30
Stroke or TIA in Other Territory	10 (33.3%)
Migraine	1 (3.3%)
Ophthalmological (non-vascular) Symptoms	2 (6.7%)
Trauma	9 (30%)
Dizziness/Vestibular Symptoms	3 (10%)
Cerebral Aneurysm	2 (6.7%)
Syncope	1 (3.3%)
Bell's Palsy	1 (3.3%)
Tracheostomy-Site Bleeding	1 (3.3%)

Legend: TIA: transient ischemic attack.
